# Characterization of Recombinant Human Type II Collagen from CHO Cells, Functional Assessment of Chondrocytes and Alleviation of Cartilage Degeneration

**DOI:** 10.3390/ijms262010232

**Published:** 2025-10-21

**Authors:** Chuan Wang, Zhijie Zhang, Zhengqi Zha, Chunyang Lu, Hang Wang, Long Yue, Hongping Yin

**Affiliations:** School of Life Science and Technology, China Pharmaceutical University, Nanjing 210009, China

**Keywords:** recombinant collagen, structure analysis, functional characteristics, cartilage repair

## Abstract

Type II collagen (Col2), a crucial structural protein in hyaline cartilage, is essential for cartilage integrity and facilitating injury repair. However, research on recombinant type II collagen still faces many challenges, such as structure and yield, which limit the application of recombinant Col2 in biomedical fields. In this study, we achieved high-yield expression of full-length human Col2 (rhCol2) in CHO cells. The physical and chemical properties of rhCol2 were very close to native Col2, including molecular weight, triple helix structure, thermal stability and self-assembly capacity. Functional assays of primary chondrocytes have demonstrated that rhCol2 can effectively promote chondrocyte proliferation and increase the expression levels of cartilage-specific genes (Col2a1, Aggrecan, and Sox-9). Moreover, a cartilage defect model was surgically created in SD rats demonstrated that rhCol2 significantly enhanced cartilage repair, and the severity of the defect was assessed through histological and micro-CT analyses. Human chondrocytes were utilized to compare the effects of different collagens and verified through a series of functional experiments. In conclusion, these findings indicate that rhCol2 is an effective biomaterial and is expected to promote the application of recombinant collagen in the field of cartilage repair.

## 1. Introduction

Type II collagen (Col2) is a fibrous type of collagen that is widely present in hyaline cartilage [1]. Col2 combines with hyaluronic acid (HA) [2], chondroitin sulfate (CS) [3] and other molecules to form the cartilage matrix, which helps cartilage resist mechanical pressure, bear loads and absorb impacts. Thus, Col2 plays a crucial role in maintaining the structural integrity and biomechanical properties of cartilage. Each peptide chain of human Col2 consists of 1487 amino acids, including a signal peptide, N-terminal propeptide, N-terminal telopeptide, triple helix domain, C-terminal telopeptide and C-terminal propeptide. The three α peptide chains form a stable triple helix structure [4].

Col2 is synthesized mainly by chondrocytes, and the synthesis and degradation of Col2 are in a dynamic balance. The increasing prevalence of sports activities and the aging global population have contributed to the rising annual incidence of joint cartilage injuries, strains and degenerative diseases [5,6]. However, due to the limitations of cartilage tissue, such as low-cell-density tissue without blood vessels, nerves or lymphatics, cartilage exhibits limited regenerative capacity. Consequently, cartilage injuries are difficult to treat and may lead to osteoarthritis of the joint [7]. The treatment of cartilage injury is difficult in orthopedics; the important aspects of cartilage repair involve the promotion of chondrocyte proliferation and the synthesis of the extracellular matrix [8]. In vitro chondrocyte cultures demonstrate enhanced viability and differentiation maintenance when supplemented with soluble collagen [9] or cultured within collagen-based scaffolds [10]. Moreover, Col2 specifically exhibits superior efficacy in maintaining differentiation capacity and biosynthetic functions [11,12].

Currently, natural Col2 is extracted primarily from terrestrial and marine organisms, including the joint cartilage of porcine [13], bovine [14], and chicken sternums [15,16], as well as from squids [17], jellyfish [18], and cartilaginous fish [19]. However, Col2 extracted from different species shows subtle structural differences due to variations in the raw materials used. Moreover, there may also be variations between different batches. These animal-derived types of Col2, also known as undenatured Col2, may have immunogenicity and other safety and religious issues [20]. Therefore, the recombinant expression of Col2 is of vital importance for producing stable, safer and more reliable supplies of Col2, which is conducive to ensuring its effective application in various fields.

In recent years, with the development of recombinant collagen technology, research on recombinant Col2 has made significant progress [21]. Researchers have used various systems to express recombinant Col2, including yeast [22,23,24], and baculovirus–silkworm [25] and Mammalian cell [26] systems. However, owing to the limitations of these expression systems, recombinant collagen studies concentrate on the production of collagen fragments. Consequently, recombinant expression of full-length Col2 remains relatively limited and typically yields low quantities.

Here, we describe the preparation of full-length human Col2 through CHO cells to meet various application needs in cartilage repair. The purpose of this research was to express full-length recombinant Col2 with biological activity. Therefore, CHO cells were selected to express recombinant human Col2, and the yield was greatly increased by screening signal peptides and selecting high-yield monoclonal cell lines. The final yield could preliminarily meet the needs of large-scale production. Subsequently, ion exchange columns were used for capture, followed by pepsin digestion for further purification, resulting in high-purity pepsin-soluble rhCol2 (PSC2). Through physical and chemical analyses, including peptide mapping, amino acid composition, isoelectric point analysis, FTIR and CD spectra, as well as TEM and SEM imaging, PSC2 was confirmed to be highly similar to natural Col2. The use of chondrocytes to evaluate the function of PSC2 revealed that PSC2 can promote chondrocyte proliferation and migration and maintain the characteristics of chondrocytes, thereby helping to maintain their biological characteristics. In addition, in a rat cartilage defect model, PSC2 was confirmed to be beneficial for cartilage defect repair and is expected to become an active substance in this process.

## 2. Results

**Figure 1:**
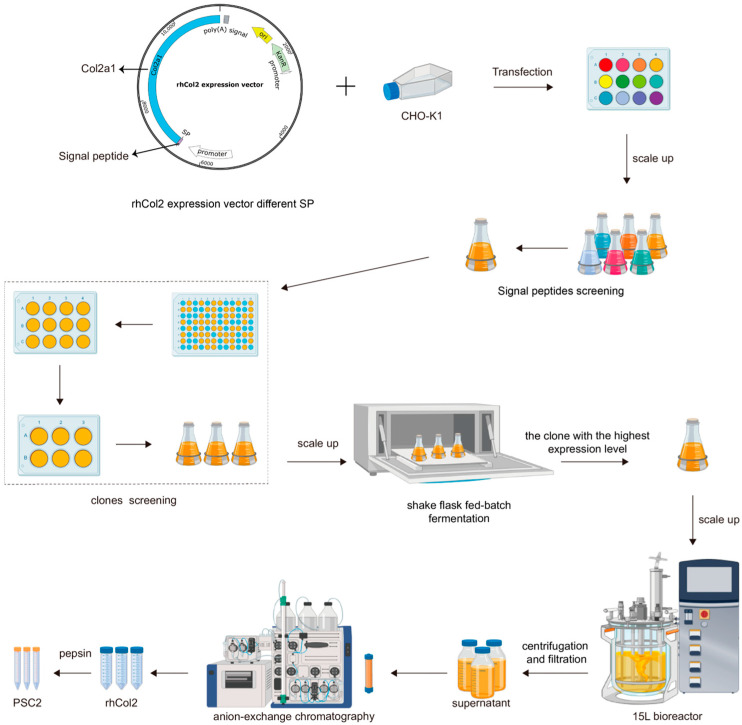
A flow sheet of recombinant human type II collagen (rhCol2) expressed by CHO cells, leading to pepsin soluble type II collagen (PSC2) obtained by anion-exchange chromatography and pepsin digestion.

Recombinant human type II collagen (rhCol2) was produced in CHO cells using signal peptide screening to enhance yield, scaled via 50 L fermentation, and purified sequentially with anion-exchange chromatography and pepsin digestion. A flowchart is shown in Figure 1.

### 2.1. Evaluation of Various Signal Peptides

**Figure 2:**
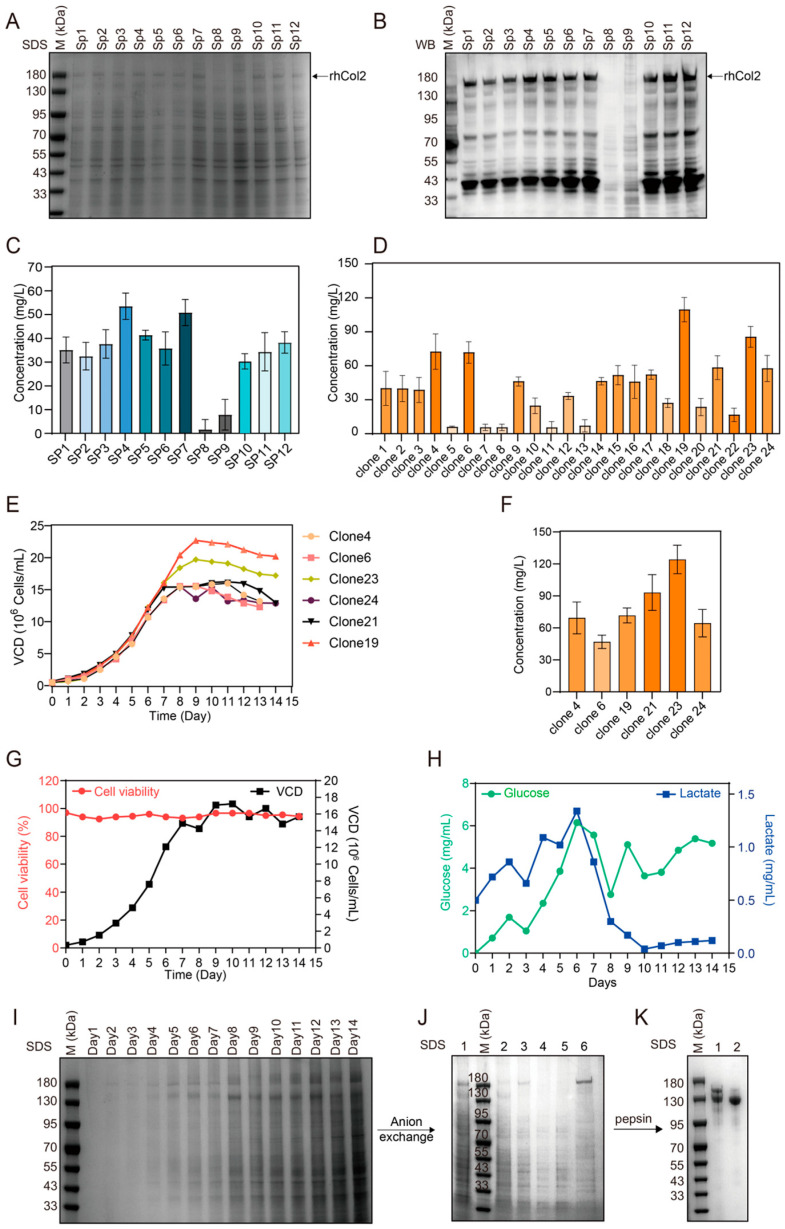
High-level expression and purification of rhCol2. (**A**) SDS–PAGE analysis of rhCol2 expression with different signal peptides. (**B**) Western blot analysis of rhCol2 expression with different signal peptides. (**C**) Evaluation of the rhCol2 expression levels by ELISA (*n* = 3). (**D**) Evaluation of the rhCol2 expression levels in 24 clones. (**E**) Growth profile of rhCol2-expressing clones in shake flasks. (**F**) Evaluation of the rhCol2 expression levels of six clones expressed in shake flasks by ELISA (*n* = 3). (**G**) Plot of the cell number and cell viability of clone 23 in a batch culture bioreactor. (**H**) Lactate accumulation and glucose consumption curve of clone 23 in a batch culture bioreactor. (**I**) SDS–PAGE image of daily samples from the bioreactor. (**J**) SDS–PAGE analysis of rhCol2 purified by anion-exchange chromatography. Lane M: protein marker; Lane 1: fermentation supernatant; Lane 2: flow-through after sample loading; Lanes 3–4: column wash fractions; Lane 5: elution with 10% elution buffer; Lane 6: elution with 45% elution buffer. (**K**) SDS–PAGE pattern of PSC2 treated with pepsin for different durations (Lane 1: 4 h; Lane 2: 8 h).

Signal peptides strongly affect the synthesis and secretion of recombinant proteins expressed in mammalian cells. To identify efficient signal peptides for enhancing recombinant human type II collagen (rhCol2) expression in CHO cells, we screened 11 secretory signal peptides. The potential of these compounds to increase the expression of rhCol2 in CHO cells was tested. Stable cell lines were established to evaluate the expression of rhCol2 (Figure 2A,B). Signal peptides 8 and 9 were unfavorable for rhCol2 expression, with almost no product detected in the supernatant. The ELISA results indicated that the human Col3a1 signal peptide and the mouse Col3a1 signal peptide were the most effective at increasing rhCol2 expression, reaching concentrations of 55.78 and 53.06 mg/L, respectively (Figure 2C). Compared with those of the wild-type signal peptide, the expression levels increased by 52.19% and 44.76%, respectively. The human type III collagen signal peptide (SP4) demonstrated the most significant enhancement in the yield of recombinant human type II collagen (rhCol2). Based on this result, Col3a1 signal peptide was chosen for further investigation.

### 2.2. High-Level Expression and Purification of rhCol2

High-yielding monoclonal cell lines were selected from stable CHO cell lines expressing rhCol2 with the human Col3a1 signal peptide. A total of 120 monoclonal cells were selected and analyzed via ELISA, and data from the top 24 monoclonal cells in terms of expression levels are presented (Figure 2D). Among these, six clones with relatively high expression levels were chosen for shake flask fed-batch comparison. After 14 days of culture (Figure 2E), the concentrations of rhCol2 in the culture media were compared, and the clone with the highest expression level achieved a yield of 139.58 mg/L (Figure 2F). The clone was subsequently used for large-scale cultivation in a bioreactor. During the process, the dissolved oxygen content, pH and other parameters were adjusted (Figure 2G–I), and the yield of rhCol2 reached 188.73 mg/L, which was approximately 35.21% greater than that of the shake flask fermentation.

To obtain rhCol2, anion-exchange chromatography was employed to capture rhCol2 from the culture medium. No significant amount of rhCol2 was detected during the sample loading and washing steps, and rhCol2 was eluted with 45% elution buffer. The SDS–PAGE results revealed that the collected fraction contained a large amount of the target protein. However, the purity still did not reach the requirements (Figure 2J). After enzymatic digestion for 4 h, the molecular weight of the single chain of rhCol2 decreased to approximately 130–150 kDa. After enzymatic digestion for 8 h, the molecular weight of the rhCol2 single chain decreased to 130 kDa, with no further changes observed with longer digestion times (Figure 2K). The target product was concentrated with a dialysis bag to remove pepsin and stored at 4 °C. This portion of the collagen was called pepsin-soluble rhCol2 (PSC2). The recovery yield of PSC2 was approximately 71.25% after two steps of purification.

### 2.3. Characterization of PSC2

**Figure 3:**
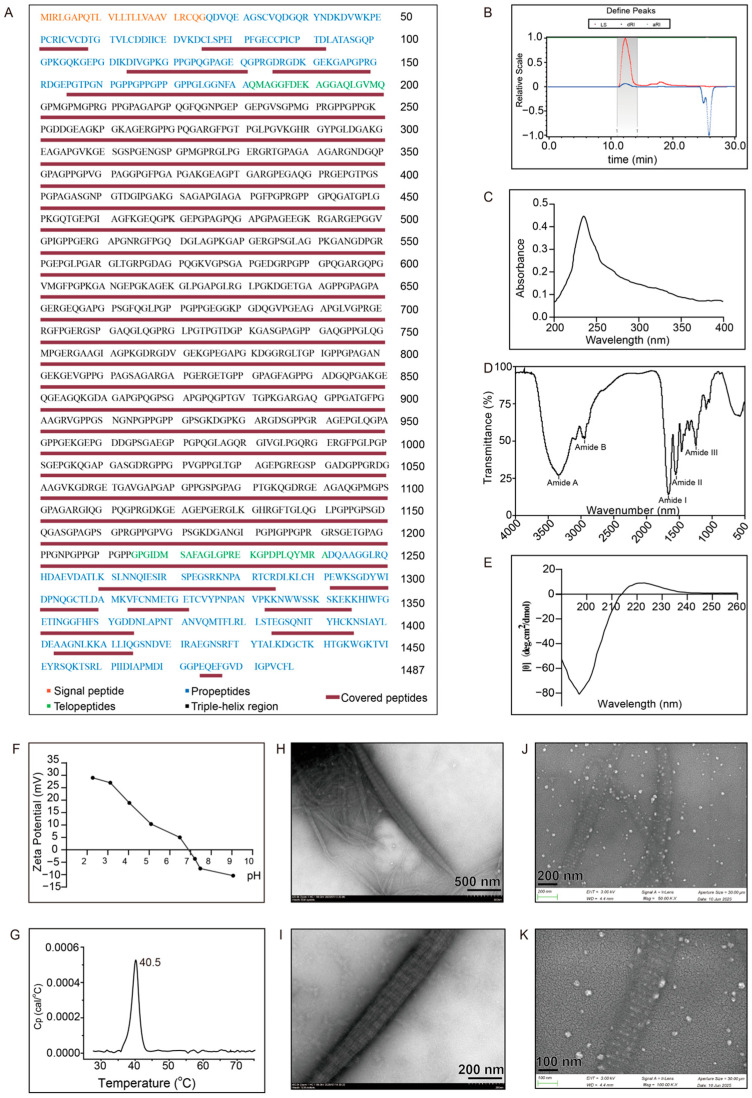
Characterization of PSC2. (**A**) the theoretical amino acid sequence of human type II collagen (the amino acids presented in red represent the signal peptide, those presented in blue represent propeptides, those presented in green represent telopeptides and those presented in black represent triple-helix regions; the covered peptides detected in the experiments are underlined); (**B**) determination of the molecular weight of PSC2 in the nonreducing state using a multiangle laser scattering detector; (**C**) UV ab-sorption spectra of PSC2; (**D**) FTIR spectra of PSC2; (**E**) CD spectra of PSC2; (**F**) zeta potential of PSC2 at various pH values; (**G**) differential scanning calorimetry (DSC) thermogram of PSC1; (**H**,**I**) TEM images of the microscopic structure of neutral PSC2 with different dimensions; (**J**,**K**) SEM images of the microscopic structure of neutral PSC2 with various dimensions.

Peptide mapping was conducted to verify PSC2 (Figure 3A). The sequence coverage confirmed by the MS data was 86.35%, with complete coverage (100%) of the triple-helix region. Based on the analysis of the fragments, the lost fragments were concentrated in the propeptide part, which is related to the fact that the propeptide part is not resistant to pepsin. The average molar masses were calculated from the scattering intensity (Figure 3B). The number-average molecular weight (Mn) and weight-average molecular weight (Mw) were determined to be 3.148 × 10^5^ and 3.157 × 10^5^, respectively. The amino acid analysis results revealed that PSC2 contained 33.1% glycine, 11.50% proline, and 9.10% hydroxyproline (Table 1). The proportion of hydroxyproline was slightly lower than that of native type II collagen. The UV absorption spectra of PSC2 revealed that PSC2 presented a maximum absorption peak at 228 nm (Figure 3C). The absence of obvious absorption at 280 nm indicated that there were fewer noncollagen components in PSC2. FTIR has been used to study changes in the secondary structure of collagen. The spectral results revealed that PSC2 contained five main absorption bands: amide A (3328 cm^−1^), amide B (2928 cm^−1^), amide I (1652 cm^−1^), amide II (1541 cm^−1^), and amide III (1241 cm^−1^) (Figure 3D). These peaks are also similar to the characteristic FTIR peaks of human type II collagen. The CD spectrum of PSC2 showed a maximum positive peak at 220 nm, a maximum negative peak at 195 nm, and an absorption ratio (Rpn) of 0.105 for the maximum positive peak and negative peak (Figure 3E). These results indicated that PSC2 has a relatively stable triple helix structure. The zeta potentials of the PSC2 solutions at different pH values were measured (Figure 3F). The zero surface net charge of PSC2 was observed at pH 6.8, with zero net charge at its isoelectric point (pI). Differential scanning calorimetry (DSC) was used to investigate the thermal denaturation (T_max_) of PSC2 (Figure 3G). Endothermic peaks, with a maximum temperature (T_max_) of 40.5 °C, were observed for PSC2 rehydration in acetic acid. Under neutral conditions, PSC2 could form a fibrous structure and precipitate from the solution. TEM revealed that the diameters of these fibrous structures ranged from several tens of nanometers to several hundreds of nanometers (Figure 3H,I). SEM images were taken at various magnifications and revealed that these fibers displayed rotational characteristics (Figure 3J,K). The characteristic D-band structure of the assembled fibrils was consistently observed in both TEM and SEM analyses.

### 2.4. Effects of PSC2 on Rat Chondrocytes

**Figure 4:**
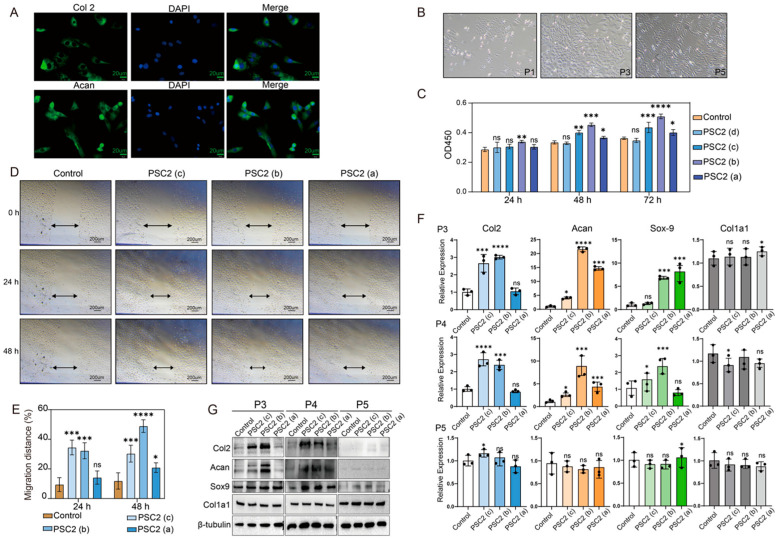
(**A**) IFA analysis of the protein expression levels of Col2and Aggrecan in rat chondrocytes. Blue: DAPI nuclear stain. Scale bar: 20 µm (**B**) Microscopy images of chondrocytes at passages 3, 4 and 5. (**C**) Statistical analysis of rat chondrocytes proliferation promoted by PSC2 (*n* = 6, * indicates the difference between each group and the control group; * *p* < 0.05, ** *p* < 0.01, *** *p* < 0.001 and **** *p* < 0.0001; ns, not significant). (**D**) Micrographs of rat chondrocytes migration at various times. (**E**) Statistical analysis of rat chondrocyte migration promoted by PSC2 (*n* = 5, * indicates the difference between each group and the control group; * *p* < 0.05, *** *p* < 0.001 and **** *p* < 0.0001; ns, not significant). (**F**) qRT–PCR analysis of the expression levels of Col2a1, Aggrecan, Sox-9, and Col1a2 in chondrocytes at passages 3, 4 and 5 treated with various concentrations of PSC2 (*n* = 3, * indicates the difference between each group and the control group; * *p* < 0.05, *** *p* < 0.001 and **** *p* < 0.0001; ns, not significant). (**G**) Western blots of Col2, Aggrecan, Sox-9 and Col1a2 in chondrocytes at passages 3, 4 and 5 treated with different concentrations of PSC2.

Immunofluorescence staining was performed to identify the cells isolated from the tissue (Figure 4A). The isolated cells expressed Col2 and Aggrecan and were identified as chondrocytes. Rat chondrocytes were expanded in monolayer cultures for up to six passages [27], and some cells exhibited the characteristics of fibroblasts (Figure 4B). The effects of PSC2 on the proliferation of rat chondrocytes were first evaluated (Figure 4C). After 24 h of culture, the proliferation rate of cells cultured on plates precoated with 3.3 µg/cm^2^ significantly increased. After 48 h of culture, the proliferation rates of cells on plates precoated with 1.0, 3.3 and 10.0 µg/cm^2^ PSC2 were greater than those of the blank group. Moreover, the number of cells cultured on plates precoated with 0.33 µg/cm^2^ PSC2 was not significantly different from that of the blank group. After 72 h of culture, except for the 0.33 µg/cm^2^ precoated group, all the other groups also presented significantly increased cell viability. Based on the results of rat chondrocyte proliferation, we evaluated the effect of PSC2 on the migration of rat chondrocytes (Figure 4D,E). After culturing for 24 h, the relative migration rates of the PSC2 pretreated with 1.0 and 3.3 µg/cm^2^ were 34.49% and 32.29%, respectively, which were significantly greater than that of the blank control group (9.45%). After culturing for 48 h, the relative migration rates of the PSC2 pretreated with 1.0, 3.3 and 10.0 µg/cm^2^ were 32.33%, 48.92% and 20.91%, respectively, which were higher than that of the blank control group (11.87%). These experimental results indicate that PSC2 can promote the proliferation and migration of rat chondrocytes and that these effects are dose-dependent within a certain concentration range. The effects of PSC2 on chondrocyte dedifferentiation [28] were finally assessed. The gene and protein expression levels of Col2a1, Aggrecan, Sox-9 and Col1a1 were measured (Figure 4F,G). In P3 chondrocytes, the mRNA levels of *Col2* cultured on PSC2 precoated plates at concentrations of 1.0 and 3.3 µg/cm^2^ were 2.6, and 3.1 times greater than those in the blank group, respectively. The mRNA levels of *Acan* were 7.7, 21.52, and 14.66 times greater than those in the blank group, whereas the mRNA levels of *Sox-9* were 2.63, 6.83, and 6.51 times greater than those in the blank group. The protein expression results were consistent with the trend of the mRNA levels. During chondrocyte culture expansion, the downregulation of Col2 and Aggrecan indicates the process of chondrocyte dedifferentiation. With increasing passages, here was no significant difference in the expression levels of the above genes and proteins compared with those in the blank group. These findings indicate that PSC2 can promote the functions and activities of chondrocytes.

### 2.5. PSC2 Promotes In Vivo Cartilage Regeneration

**Figure 5:**
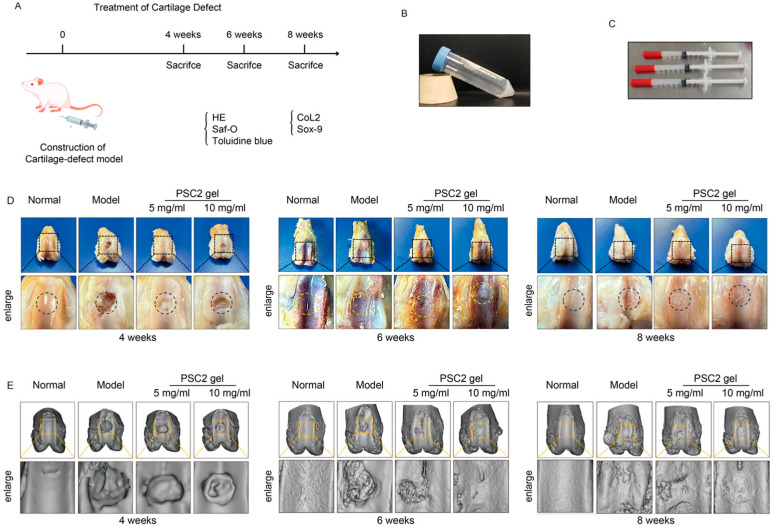
Evaluation of PSC2 activity in rat chondrocytes: (**A**) experimental procedure for the use of PSC2 gel for the formation of cartilage tissue in vivo; (**B**) image of neutral PSC2; (**C**) PSC2 gel in syringes for injection; (**D**) representative images of gross observations of cartilage defects treated with PSC2 gel at 4, 6 and 8 weeks postoperation in vivo in rats; (**E**) micro-CT three-dimensional (3D) imaging of the knee joints of rats treated with PSC2 gel at 4, 6 and 8 weeks.

The efficiency of PSC2 for cartilage regeneration applications was further verified (Figure 5A). PSC2 gel was generated and was white and gelatinous and exhibited favorable injectable properties for potential therapeutic applications (Figure 5B,C). At 4 weeks post-operation, gross morphological examination revealed that the defect repair speed of the model group was much lower than that of the PSC2 treatment group (Figure 5D). Micro-CT analysis revealed significant differences in knee joint structure among the different treatment groups. Three-dimensional micro-CT images (Figure 5E) revealed increased osteophyte formation in the model group, whereas the PSC2 treatment groups presented less osteophyte formation at 6–8 weeks postoperation.

**Figure 6:**
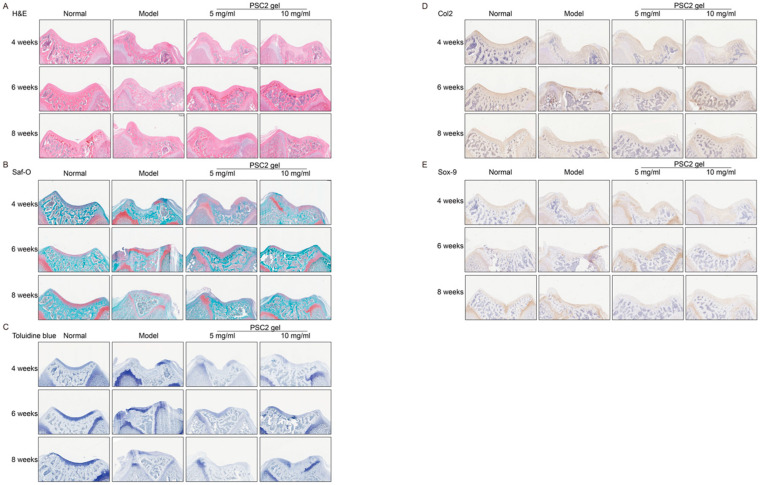
In vivo PSC2 gel viability evaluation. (**A**) Representative images of HE staining of rat cartilage. (**B**) Representative images of safranin-O/fast green staining of rat cartilage. (**C**) Representative images of toluidine blue staining of rat cartilage. (**D**) Immunohistochemical staining for Col2 in rat cartilage. (**E**) Immunohistochemical staining for Sox-9 in rat cartilage.

Histological evaluations revealed that the degree of cartilage repair in the model groups was lower than those in the PSC2-treated groups and that the cartilage in the PSC2 treatment groups was smoother than that in the model group (Figure 6A). Moreover, the repair tissue in the model group displayed only slight matrix staining for sGAG and Col2. Comparatively, the defects in the PSC2 treatment groups presented relatively strong matrix staining for sGAG and Col2 (Figure 6B,C). Immunohistochemical analysis revealed that fewer newly formed tissue cells expressed the cartilage cell markers Col2 and Sox-9 in the model group (Figure 6D,E). In contrast, in the PSC2 treatment groups, the number of Col2- and Sox-9 positive cells increased, and these cells were initially identified as newly formed chondrocytes. In conclusion, PSC2 significantly repaired cartilage damage, significantly regulating cell proliferation and the production of the cartilage matrix.

### 2.6. Effects of Different Types of Collagen on Human Chondrocytes

**Figure 7:**
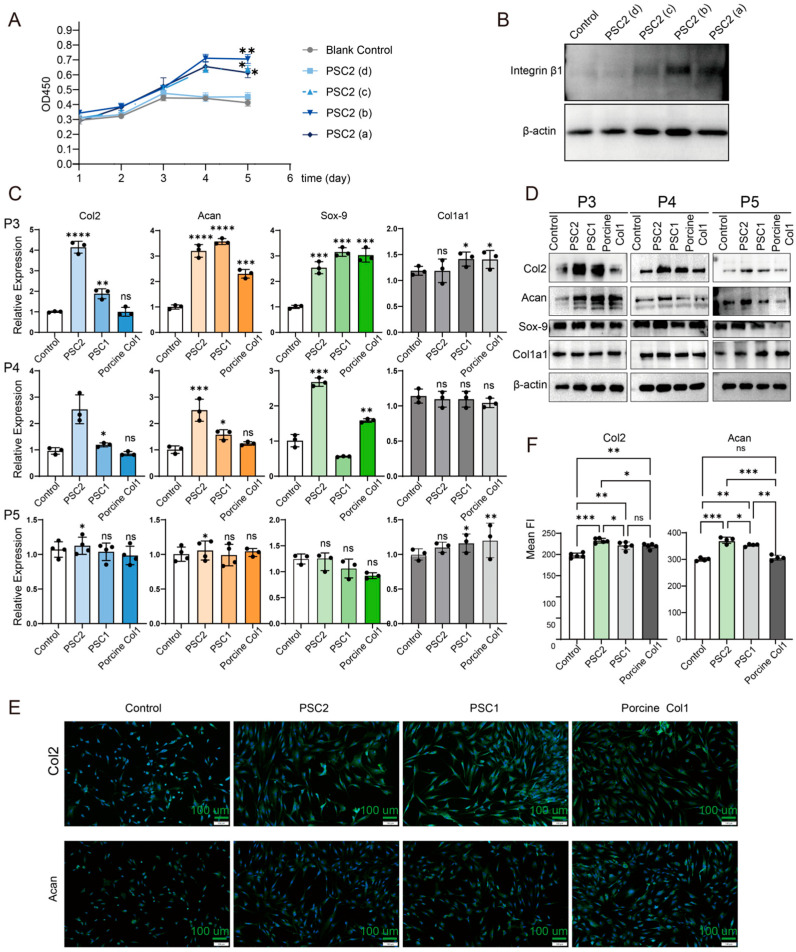
Evaluation of the activity of different collagens in human chondrocytes. (**A**) Effects of PSC2 on human chondrocyte proliferation over 5 days. (**B**) Protein expression of integrin β1 in human chondrocytes after treatment with different concentrations of PSC2. (**C**) qRT–PCR analysis of the expression levels of Col2a1, Aggrecan, Sox-9 and Col1a1 in human chondrocytes at passages 3, 4 and 5 after treatment with PSC2, PSC1 or porcine col1 (*n* = 3, * indicates a difference between each group and the control group; * *p* < 0.05, ** *p* < 0.01, *** *p* < 0.001 and **** *p* < 0.0001; ns, not significant). (**D**) Western blots of Col2, Aggrecan, Sox-9 and Col1a1 in human chondrocytes at passages 3, 4 and 5 treated with PSC2, PSC1 or porcine col. (**E**) IFA analysis of the protein expression levels of Col2 and Aggrecan in P6 human chondrocytes. (**F**) Quantification of the immunofluorescence results (*n* = 5, * indicates a difference between each group and the control group; * *p* < 0.05, ** *p* < 0.01 and *** *p* < 0.001; ns, not significant).

The effects of PSC2 on the proliferation of human chondrocytes were first evaluated (Figure 7A), and the experimental duration was extended to five days. The human chondrocytes in the DMEM control group proliferated slowly and almost stopped proliferating after 3 days. Moreover, the cell proliferation rates of the groups precoated with 1.0, 3.3 or 10.0 µg/cm^2^ PSC2 were significantly greater than those of the DMEM control group (*p* < 0.01), with the 3.3 µg/cm^2^ group showing the greatest effect. These findings indicate that PSC2 can significantly promote chondrocyte proliferation. Furthermore, Western blot analysis revealed that PSC2 treatment increased the expression level of integrin β1 (Figure 7B), a common collagen receptor critical for promoting human chondrocyte proliferation [29].

Three types of collagens were applied to precoated cell plates at the same concentration to investigate their impact on human chondrocyte dedifferentiation. The effects of PSC2, PSC1, and porcine collagen on human chondrocytes were evaluated using qRT–PCR and Western blot (Figure 7C,D). The results indicated that three kinds of collagen could maintain the characteristics of chondrocytes. In P4 and P5 human chondrocytes, the PSC2-treated group exhibited significantly higher mRNA levels of Col2 and Aggrecan compared to the control group (*p* < 0.05), and a slower rate of protein decrease than the PSC1 and porcine collagen groups. The results of the immunofluorescence analysis of the last passage of chondrocytes also confirmed this phenomenon (Figure 7E,F). Overall, the results demonstrate that PSC2 was the most effective of the treatments tested in maintaining chondrocyte marker genes. Collectively, these findings indicate that PSC2 is more effective than either PSC1 or porcine collagen at sustaining the stability and function of human chondrocytes.

## 3. Discussion

Research on the expression of recombinant collagen has made significant progress in recent years [30]. However, most current studies focus on the expression of collagen fragments in host cells, such as *Escherichia coli* and yeast. CHO cells are among the most widely used mammalian cell lines in biopharmaceuticals. CHO cells can express full-length collagen with a complete triple-helix structure. However, due to the lower production efficiency of CHO cells, they express proteins with a greater degree of modification.

Common methods to increase the yield of CHO cells include optimizing expression vectors and signal peptides, obtaining high-yield monoclonal cells, and optimizing fermentation conditions. In this study, we focused on optimizing the expression of rhCol2 by screening various signal peptides. Our results demonstrated that not all signal peptides are effective for rhCol2 expression. However, the final yield was still lower than the yield of rhCol1a1. These findings indicate that different types of collagens have different expression efficiencies in CHO cells. Future research can explore other optimization strategies, such as screening domains more suitable for recombinant expression of collagen or modifying the metabolic pathways of CHO cells using knockout techniques. These approaches might enhance the expression efficiency of recombinant collagen.

The triple helix structure is essential for collagen function. PSC2 exhibited relatively high hydroxyproline content, a critical factor for triple helix formation. FTIR analysis revealed that PSC2 has the same 5 absorption peaks as natural Col2, namely, amide bands (A, B, I, II, and III). CD spectroscopy revealed that PSC2 has a maximum positive peak at 220 nm and a maximum negative peak at 195 nm. The denaturation temperature detected by DSC was 40.5 °C, which indicates that PSC2 can remain stable at body temperature. These results suggest that PSC2 has a complete triple helix structure that is similar to the structure of natural Col2. Collectively, our results demonstrate the successful expression of rhCol2 in CHO cells, with enhanced yield sufficient for preliminary applications.

To evaluate the ability of PSC2 in cartilage repair requires chondrocytes. The culture conditions of primary chondrocytes are relatively strict, and with increasing passage number, they gradually undergo dedifferentiation, resulting in significant changes in their biological characteristics. PSC2 has poor solubility in neutral solution in the culture medium; therefore, to evaluate the activity of PSC2, it was precoated on a cell culture plate. The results showed that PSC2 had obvious proliferative and migratory effects on primary rat chondrocytes within a certain concentration range, whereas an excessively high coating concentration may affect the extracellular environment of chondrocytes, resulting in a decrease in the stimulating effect on chondrocytes. Chondrocytes can secrete Col2 and proteoglycans to form the cartilage matrix, which is crucial for the structure and function of cartilage. This study analyzed the effects of PSC2 on chondrocyte characteristics through qRT–PCR and Western blotting. The results showed that under the direct action of PSC2, the process of downregulating genes such as Col2 and Aggrecan in chondrocytes was slowed. Although the trends in the mRNA and protein expression levels were not completely synchronous, in general, PSC2 was helpful in maintaining the normal state of chondrocytes, which was similar to reports from other researchers. This positive effect may extend to the auxiliary treatment of diseases such as osteoarthritis.

Collagen can self-assemble into an ordered network structure under suitable conditions [31], which is conducive to cell proliferation, migration, and adhesion [32]. Additionally, owing to its good biocompatibility and degradability, it has enormous application prospects in regenerative medicine and tissue engineering [33]. In this study, we explored the conditions for the self-assembly of PSC2 into hydrogels. When incubated at 37 °C for several hours, a substance similar to a gel could initially form, which may be related to the fact that its molecules can form a network structure. However, the mechanical strength of the hydrogels formed by PSC2 self-assembly under the current experimental conditions is still insufficient to meet the requirements of material applications, especially when the material needs to be colonized near defect sites. In subsequent explorations, the self-assembly experimental conditions can be further optimized, or the cross-linking reactions can be increased to produce hydrogels with greater mechanical strength [34]. We will also attempt to conduct cross-linking reactions with large molecules such as chondroitin sulfate or glucosamine to make composite materials, thereby improving the effect of cartilage repair.

Cartilage regeneration has always been a difficult problem in clinical medicine. Cartilage defects are often closely related to the occurrence of degenerative joint arthritis (osteoarthritis, OA). In recent years, various cartilage repair materials have emerged in succession. Currently, Col1 and Col3 are widely used in articular cartilage repair [35]. Meanwhie, the repair materials developed from Col2 can more effectively simulate the microenvironment [36] of natural cartilage. Therefore, developing cartilage repair materials using safe and effective Col2 has great potential. Future research will further optimize the preparation process and application methods of PSC2, develop more mature cartilage repair materials, and improve their application value in clinical practice, providing new strategies for the prevention and treatment of joint diseases.

## 4. Materials and Methods

### 4.1. CHO Cell Culture

The CHO K1-cell line was purchased from CellCook Biochemical Co., Ltd. (Guangzhou, China), adapted to suspension culture and grown in VegaCHO medium (OPM Biosciences, Shanghai, China). The cultures were maintained at 37 °C in a 5% CO_2_ incubator with shaking at 120 rpm.

### 4.2. Expression of rhCol2

#### 4.2.1. Vector Construct

The cDNA encoding human type II collagen α1 (amino acid residues 1 to 1487) was synthesized by GENEWIZ and inserted into the pCMV3 expression vector. To assess the secretion efficiencies of the different signal peptides (SPs), as listed in Table 2, each signal peptide, oligonucleotides containing a Kozak consensus sequence and the respective sequence coding for the signal peptide were synthesized. These SPs were used to replace the wild-type signal peptide in the rhCol2 construct, and 12 different rhCol2 plasmids were subsequently constructed.

#### 4.2.2. Transfection and Stable Expression

For transient expression, CHO K1 cells were seeded in a 6-well plate one day before transfection. The cells (1 × 10^6^) were transfected with 10 µg of linearized plasmid DNA using electroporation. Forty-eight hours post transduction, to obtain stable expression, the medium was replaced with fresh VegaCHO Plus medium containing 0.2–0.5 mg/mL hygromycin. The selection medium was changed every 3–4 days for 2 weeks. Stably transfected cells were expanded for rhCol2 quantitation

#### 4.2.3. Screening of the Optimal Signal Peptide

To screen the optimal signal peptide, stable cell lines (pooled cells) were inoculated into 125 mL flasks at 5 × 10^6^ cells/mL with 25 mL for suspension culture. The flasks were incubated at 37 °C in 5% CO_2_ with shaking at 120 rpm for cultivation (Zhichu Instruments, Shanghai, China). The cell density and viability were recorded with a Countess™ 3 (Thermo Fisher, Waltham, MA, USA). When the cell viability decreased to 80%, the supernatant was collected, and the rhCol2 expression levels were quantified with an ELISA kit (Solarbio SEKH-0628).

#### 4.2.4. Screening for High-Yield Cloning

High-yield clone cell lines were selected from the mixed cell lines via limited dilution. The mixed cell lines were adapted to QuaMono™ Plus CHO medium (QuaCell Biotechnology Zhongshan, China). The cells were subsequently diluted and inoculated into a 96-well plate. The wells containing one single cell were identified and marked for expansion. These clones were subsequently inoculated into 24-well plates at a density of 2 × 10^5^ cells/mL. After 7 days of culture, the supernatant was collected and analyzed by ELISA. Clones with relatively high expression levels were inoculated into flasks for further screening. The shaking flask fermentation conditions refer to the recommended usage of the culture medium. In brief, the cells were inoculated into 50 mL of VegaCHO Plus Medium at a density of 5 × 10^6^ cells/mL and batch fed with the feed medium CDF056 and CDFS36 (OPM Biosciences, Shanghai, China). The culture supernatant was harvested for ELISA detection of rhCol2.

#### 4.2.5. Production of rhCol2 in a Bioreactor

The best clone was used for scaling up and subjected to rhCol2 production in a 15 L bioreactor (Tchuyee, Shanghai China). The culture conditions followed the shaking flask fermentation process. The dissolved oxygen levels were maintained above 30% by adjusting the agitation rate and sparging with air and oxygen. The pH was maintained within the range of 7.0–7.2. Glucose and lactate levels were monitored and controlled to optimize cell growth and productivity. The cell density and viability were determined.

### 4.3. Purification of rhCol2

The culture medium containing rhCol2 was centrifuged to remove the cells. The clarified medium was then loaded onto an anion-exchange chromatography column (HiTrap Capto Q, Cytiva Lifescience, Marlborough, NH, USA). The column was washed with 20 mM phosphoric acid buffer (pH 6.5), and the protein was eluted in a stepwise fashion with 20 mM phosphoric acid buffer containing 0.1, 0.45, and 1 M sodium chloride (pH 6.5).

High-purity protein was obtained. The fractions eluted containing rhCol2 were collected, and the pH was adjusted with hydrochloric acid (pH 2). Then, 0.1% pepsin (by mass) was added to the fraction, which was incubated for 8 h at 4 °C. The separation of pepsin-solubilized rhCol2 (PSC2) and pepsin was performed using a dialysis bag. Dialysis was carried out for 16–24 h with three buffer changes. The purified PSC2 was subsequently stored at 4 °C.

### 4.4. SDS–PAGE and Western Blot Analysis

The CHO cell culture medium was centrifuged to obtain the supernatant for the detection of rhCol2. The supernatant was separated on 8% SDS–PAGE gels under reducing conditions and subsequently visualized by Coomassie blue staining or Western blotting. Western blot assays were performed according to standard protocols. Proteins were transferred onto PVDF membranes, blocked with 5% nonfat milk in TBST (20 mM Tris-HCl, 150 mM NaCl, 0.1% Tween 20), and probed with primary antibody (A1560, ABclonal, Wuhan, China, 1:2000) overnight at 4 °C. The membranes were subsequently incubated with secondary antibody (33701ES60, Yeasen, Shanghai, China, 1:5000) for 60 min at room temperature.

Separately, for the chondrocytes, total protein was extracted from the cell culture plates with protein lysis buffer, and the concentrations of the protein samples were determined using a BCA assay kit. The protein samples were separated to SDS–PAGE and Western blot analysis using the same procedure as described in the previous section for the CHO cell supernatant. Primary antibodies used for analysis included anti-Col2a1 (A1560, ABclonal; 1:2000), anti-Aggrecan (ab3778, abcam; 1:2000), anti-Sox-9 (YP-Ab-15333, UpingBio, Hangzhou, China; 1:2000), anti-Col1a1 (A16891, ABclonal; 1:2000), and anti-Sox-9 (YP-Ab-17031, UpingBio; 1:3000). The HRP-conjugated β-actin (30103ES60, Yeason; 1:4000) and HRP-conjugated β-Tubulin (30303ES10, Yeason; 1:4000) were also used.

### 4.5. Peptide Mapping Analysis

Peptide mapping analysis of PSC2 was performed to compare the amino acid sequence of human type II collagen. In brief, 1.0 mg of PSC2 was denatured at 100 °C for 30 min. After that, 20 μg of trypsin was added and incubated at 37 °C for 18 h. The digestion was terminated by adding 10% formic acid. Then, 20 μg of Glu-C was added and further incubated at 37 °C for 18 h. The reaction was stopped and centrifuged to collect the supernatant for peptide mapping analysis. These peptide segments were subsequently subjected to mass spectrometry analysis and compared to the theoretical peptide masses derived from the known sequence of human type II collagen. The peptide mapping experiments were performed at the Institute of Process Engineering, Chinese Academy of Sciences, as described in the literature.

### 4.6. Amino Acid Analysis

The amino acid analysis of PSC2 was performed using high-performance liquid chromatography (HPLC) with a Shim-pack GIST C18-AQ column (5 µm, 4.6 × 150 mm), as previously described. Briefly, PSC2 was hydrolyzed with 6 M hydrochloric acid at 110 °C for 24 h. The hydrolysate was filtered and then analyzed using the Agilent amino acid derivatization protocol (Document 5991-7694EN). The amino acid composition was determined according to standard amino acids.

### 4.7. SEC-MALS Experiments

The molecular weight of PSC2 was determined using SEC-MALS [39,40]. PSC2 (1 mg/mL) was prepared in running buffer. The sample was injected and analyzed with a multiangle light scattering detector and refractometer (Wyatt Technology, Santa Barbara, CA, USA). The data were collected, and the molecular weight was calculated based on the following formula (1), where *I* is the excess light scatter signal intensity measured from the MALS. The specific index dn/dc value for large proteins is 0.186 mL/g [40], and this value was also employed for the calculation of the molecular weight of PSC2.

(1) $$MW\alpha \frac{I}{{\left(\frac{dn}{dc}\right)}^{2}C}$$

### 4.8. UV–VIS Absorption Spectra

UV spectral analysis of PSC2 was performed using a Shimadzu UV-2550 (Shimadzu Corporation, Kyoto, Japan). PSC2 (1 mg/mL) was incubated in 10 mM hydrochloric acid and placed in a quartz cuvette. The UV absorption spectrum was recorded over a wavelength range of 200–400 nm at room temperature.

### 4.9. Fourier Transform Infrared Spectroscopy (FTIR)

FTIR spectral analysis of PSC2 was performed using a Fourier transform infrared spectrometer [41,42]. PSC2 samples were prepared, mixed with KBr, and pressed into pellets. The spectra were collected in the wavelength range of 500–4000 cm^−1^, with a resolution of 1 cm^−1^.

### 4.10. Circular Dichroism (CD) Spectroscopy

The secondary structure of PSC2 was analyzed using CD spectroscopy. PSC2 was prepared in 10 mM HCl at concentrations ranging from 0.5–0.6 mg/mL. Afterward, CD spectra were recorded on a CD spectropolarimeter in the wavelength range of 190–260 nm at 25 °C with a step interval of 1.0 nm. A reference spectrum containing HCl was also recorded. The CD spectra of the samples were obtained after subtracting the reference spectrum.

### 4.11. Zeta Potential Analysis

The pI of PSC2 was determined by zeta potential analysis [41]. The PSC2 samples were dissolved in hydrochloric acid to obtain a concentration of 2 mg/mL. The pH of the solutions was adjusted to 2–10 using either 1.0 M hydrochloric acid or 1.0 M NaOH. The zeta potential of the collagen solutions was measured with a Zetasizer Nano ZS90 (Malvern Instruments, Malvern, UK). The zeta potentials of the solutions obtained at all pH values were recorded, and the pI was identified as the pH at which the zeta potential was zero [42].

### 4.12. Differential Scanning Calorimetry (DSC)

To investigate the thermostability of PSC2, PSC2 (5 mg) was sealed in aluminum pans, with an empty aluminum pan used as a reference. The endothermal curves of the samples were scanned over the range of 20–80 °C at a heating rate of 1 °C min^−1^. The maximum transition temperature (Tmax) of PSC2 was estimated from the thermogram. The total denaturation enthalpy (DH) was estimated by measuring the area of the DSC thermogram.

### 4.13. Transmission Electron Microscopy (TEM)

To evaluate the surface morphology and structure of PSC2 [43], solutions of PSC2 were adjusted to neutrality using 1.0 M NaOH. Subsequently, the PSC2 solutions were loaded onto 400 mesh Formvar carbon-coated copper grids for 10 min. Afterward, the grids were washed with deionized water, and 1% sodium phosphotungstate was used to stain the collagen fibrils. The stained grids were washed with deionized water and air-dried. The samples were subsequently analyzed using Ruli TEM.

### 4.14. Scanning Electron Microscopy (SEM)

The pH of the PSC2 solution was adjusted to neutrality [43]. The PSC2 samples were subsequently dripped onto glass plates and heated at 30 °C for 60 min. Then, the samples were sputter coated with gold and examined via SEM.

### 4.15. Chondrocyte Isolation and Culture

Cartilage from rats (2–3 weeks) was used to isolate rat primary chondrocytes [44]. Briefly, cartilage tissue was cut into slices and digested with 0.25% trypsin containing 0.02% EDTA for 30 min. Tissue slices were then digested with 0.25% collagenase II in serum-free DMEM at 37 °C for 2–6 h [10]. The digested cartilage was filtered through 70-µm nylon mesh. Primary chondrocytes were collected by centrifugation at 1200 rpm for 10 min and cultured in DMEM containing 10% FBS (Thermo Fisher, Waltham, MA, USA) and 1% penicillin/streptomycin in tissue culture flasks in a CO_2_ incubator (5% CO_2_, 95% humidity) at 37 °C. The medium was changed after 48 h to remove nonadherent cells and every 3–4 days thereafter.

Human chondrocytes were purchased from Sunncell Biochemical Co., Ltd. (Wuhan, China) and were cultured in complete chondrocyte culture medium (Sunncell Biochemical Co., Ltd., Wuhan, China).

### 4.16. Immunofluorescence Assays

Immunofluorescence was used to identify primary chondrocytes [45]. The cells were fixed with 4% paraformaldehyde for 20 min to obtain immobilized cells. After being washed three times with PBS, the cells were incubated with 0.2% Triton X-100 for 5 min. The cells were incubated with anti-Col2a1 (A1560, ABclonal; 1:500), anti-Aggrecan (A12045, ABclonal; 1:100) at 4 °C overnight. Subsequently, FITC-labeled goat anti-rabbit IgG (33307ES60, Yeason; 1:100) was incubated with the antigen–antibody complex for 2 h. The cell nuclei were counterstained with DAPI. Images were acquired using a Nikon fluorescence microscope.

### 4.17. Analysis of Chondrocyte Activity

The solubility of PSC2 is relatively low in neutral solutions, and it tends to precipitate in culture medium. To investigate the effects of PSC2 on rat chondrocytes [46], PSC2 solutions at concentrations of 2, 6, 18, and 54 µg/mL were added to 96-well plates at 50 µL per well to prepare PSC2 precoated plates at concentrations of 0.3, 1.0, 3.3, and 10 µg/cm^2^, respectively. After incubation at room temperature for 4 h, the PSC2 solutions were removed, and the plates were washed three times with PBS and stored at 4 °C. Rat chondrocytes were then seeded into the 96-well cell culture plate at a density of 5 × 10^3^ cells/well. After culturing for 24, 48, or 72 h, the effects of PSC2 on cell proliferation were measured using a CCK-8 assay kit.

Human chondrocytes were cultured in complete chondrocyte culture medium, and the other conditions were the same as those used for rat primary chondrocytes. Human chondrocytes were cultured in 96-well plates precoated with 3.3 µg/cm^2^ PSC2, PSC1 [47], or porcine collagen at a density of 4 × 10^3^ cells/well. The effects of different types of collagen on cell proliferation were measured for 5 days using a CCK-8 assay kit.

### 4.18. Cell Migration

The cell migration assay was conducted in accordance with the protocol described in our previous study [47]. Rat chondrocytes were seeded into 12-well cell culture plates at a density of 3 × 10^4^ cells/well [48,49]. The 12-well plates were precoated with PSC2 at concentrations of 1.0, 3.3, and 10.0 µg/cm^2^. When the cells reached 80–90% confluence, the supernatant was removed, and a line was drawn vertically across each well. Images were captured at 0, 24 and 48 h using an inverted microscope. The migration rate was analyzed using Image-Pro-Plus software 6.0.

### 4.19. Gene Expression Analysis

Rat chondrocytes were inoculated into a 6-well cell culture plate at a density of 1 × 10^6^ cells/well. The 6-well plates were precoated with PSC2 at concentrations of 1.0, 3.3, and 10.0 µg/cm^2^. When the confluence of the cells reached 90%, the cells were subcultured on new PSC2 precoated plates at the corresponding concentrations. Real-time quantitative PCR was used to detect the expression levels of the relevant genes [46,50,51]. Total RNA was subsequently isolated using a Total RNA Isolation Kit (Vazyme RC112-01), and cDNA was synthesized using a reverse transcription kit (Vazyme R323-01). qRT–PCR was performed on a QuantStudio 3 Real-Time PCR System (Thermo Fisher, Waltham, MA, USA). The 20 µL reaction mixture contained 10 µL of 2× SYBR Green PCR Master Mix, 0.4 µL of each primer (*Col2a1*, *Acan*, *Sox-9*, *Col1a1*), and 2 µL of cDNA. Relative gene expression levels were calculated using the 2^−ΔΔCT^ method. Experiments on human chondrocytes followed an identical protocol except for the types of collagen used for coating. The primers used are listed in Table 3 and Table 4.

### 4.20. Regeneration of Articular Cartilage Defects In Vivo

To investigate the effect of PSC2 on cartilage defects. PSC2 were dissolved in 0.01 M HCl to obtain a 2 mg/mL solution. Then the pH of the PSC2 solution was adjusted to neutral (pH 7.0) using 0.2 M Na_2_HPO_4_. The precipitate was collected by centrifugation (10,000× *g*, 30 min), resuspended in sterile PBS to final concentrations of 5 mg/mL (0.5% *w*/*v*) or 10 mg/mL (1.0% *w*/*v*), and homogenized on ice at 12,000 rpm for 1 min. The resulting suspension was incubated at 35 °C for 2 h to form a physical hydrogel. Male SD rats (2–3 weeks old) were randomly divided into 4 groups: the control group (*n* = 9), model group (*n* = 12), PSC2 gel (10 mg/mL) low-dose group (*n* = 12), and PSC2 gel (10 mg/mL) high-dose group (*n* = 12). Except for the rats in the control group, cartilage defects (2 mm in diameter and 1 mm in depth) were created at the distal end of the femur [52,53], and PSC2 gel was injected into the cartilage defects. The rats were then sacrificed at specific time intervals.

### 4.21. Micro-CT Scan

Knee joint samples were collected and fixed in 4% paraformaldehyde for 48 h. Then, the samples were placed in appropriate tubes and scanned using a micro-CT system (SkyScan 1076, Kontich, Belgium) [48]. Samples were scanned using a micro-CT scanner (50 kV, 800 µA). The scanned images were reconstructed using the micro-CT system software, and three-dimensional reconstruction was performed to evaluate the ability of the PSC2 hydrogel to repair cartilage [52,53].

### 4.22. Histological and Immunohistochemical Staining

The knee joint samples were decalcified and embedded in paraffin. For histological [54] and immunohistological analyses [27], paraffin sections were cut into 4.5 µm pieces. These sections were hydrated and stained with hematoxylin and eosin (H&E), Safranin O/fast green (Saf-O), and toluidine blue. Additionally, immunohistochemical staining for Col2 and Sox-9 was performed [55]. The primary antibodies anti-Col2a1 (A1560, ABclonal; 1:200), anti-Sox-9 (YP-Ab-15333, UpingBio; 1:200), were applied at 4 °C overnight, and the samples were incubated with secondary antibodies. The stained slides were fixed and observed under a bright-field microscope for analysis.

### 4.23. Statistical Analysis

All the data are expressed as the means ± standard deviations. The means of groups were compared by analysis of variance and post hoc tests, with Tukey’s honestly significant difference test used to evaluate the statistical significance of differences in the macroscopic, histological and immunohistochemical results. *p* < 0.05 was considered statistically significant.

## Figures and Tables

**Table 1:** Amino acid analysis results.

Number	Amino Acid	PSC2	Triple Helix Region of the α1 Chain of Human Type II Collagen
1	Asp	4.85%	3.06%
2	Glu	5.53%	4.64%
3	Ser	3.40%	3.35%
4	His	0.25%	0.20%
5	Gly	33.10%	33.63%
6	Thr	0.55%	1.58%
7	Arg	5.1%	5.03%
8	Ala	15.0%	11.64%
9	Tyr	0.18%	0
10	Cys	0.36%	0
11	Val	1.70%	1.97%
12	Met	0.26%	0.69%
13	Trp	0	0
14	Phe	1.56%	0
15	Ile	1.06%	0.59%
16	Leu	2.87%	1.87%
17	Lys	2.19%	3.55%
18	Gln	1.26%	2.66%
19	Asn	0.74%	1.08%
20	Hyp	9.10%	10.95%
21	Pro	11.50%	12.33%

**Table 2:** Amino acid sequences of the signal peptides selected in this study [37,38].

Signal Peptide	Number	Amino Acid Sequence
Human Col2a1 (WT)	1	MIRLGAPQTLVLLTLLVAAVLRCQG
Human IgG	2	MGSAALLLWVLLLWVPGSNG
Modified IgG	3	MGSAALLLWVLLLWVPSSRA
Human Col3a1	4	MMSFVQKGSWLLLALLHPTI ILA
Mouse Col1a1	5	MFSFVDLRLLLLLGATALLTHG
Mouse Col2a1	6	MIRLGAPQSLVLLTLLIAAVLRCQG
Mouse Col3a1	7	MMSFVQSGTWFLLTLLHPTLILA
SA	8	MKWVTFISLLFLFSSAYS
modify SA	9	MKWVTFISLLFLFSSSSRA
AZ	10	MTRLTVLALLAGLLASSRA
Immunoglobulin heavy chain	11	MAWSPLFLTLITHCAGSWA
Immunoglobulin light chain	12	MDWTWRVFCLLAVTPGAHP

**Table 3:** qRT–PCR primers sequences for rat genes.

Gene	Primer Sequence (5′ to 3′)	Size (bp)	NCBI Reference
*Col2a1*	F: ACACCGCTAACGTCCAGATG R: TCGGTACTCGATGATGGTCT	254	NM_001414896.1
*Acan*	F: TTGGTAGGGTCTGCTTCTGG R: GATGGGCCACTTCCAATGTC	143	NM_022190.2
*Sox-9*	F: AAATTCCCAGTGTGCATCCG R: TGACGTGTGGCTTGTTCTTG	115	NM_080403.3
*Gapdh*	F: CAAGGCTGAGAATGGGAAGC R: GAAGACGCCAGTAGACTCCA	127	NM_017008
*Col1a2*	F: CTGAGGGCAACAGCAGATTC R: CAGGCGAGATGGCTTATTCG	113	NM_053356.2

**Table 4:** qRT–PCR primers sequences for human genes.

Gene	Primer Sequence (5′ to 3′)	Size (bp)	NCBI Reference
*Col2a1*	F: TGGACGATCAGGCGAAACC R: GCTGCGGATGCTCTCAATCT	244	NM_001844.5
*Acan*	F: GTGCCTATCAGGACAAGGTCT R: GATGCCTTTCACCACGACTTC	167	NM_001135.4
*Sox-9*	F: AGCGAACGCACATCAAGAC R: CTGTAGGCGATCTGTTGGGG	86	NM_000346.4
*Gapdh*	F: GGAGCGAGATCCCTCCAAAAT R: GGCTGTTGTCATACTTCTCATG	197	NM_001256799.3
*Col1a2*	F: GAGGGCCAAGACGAAGACATC R: CAGATCACGTCATCGCACAAC	140	NM_000089.4

## Data Availability

The data that support the findings of this study are available from the corresponding author upon reasonable request. Some data may not be made available because of privacy or ethical restrictions.

## References

[1] Wu Z., Korntner S.H., Mullen A.M., Zeugolis D.I. (2021). Collagen type II: From biosynthesis to advanced biomaterials for cartilage engineering. Biomater. Biosyst..

[2] Intini C., Hodgkinson T., Casey S.M., Gleeson J.P., O’Brien F.J. (2022). Highly Porous Type II Collagen-Containing Scaffolds for Enhanced Cartilage Repair with Reduced Hypertrophic Cartilage Formation. Bioengineering.

[3] Ko C.S., Huang J.P., Huang C.W., Chu I.M. (2009). Type II collagen-chondroitin sulfate-hyaluronan scaffold cross-linked by genipin for cartilage tissue engineering. J. Biosci. Bioeng..

[4] Ricard-Blum S. (2010). The Collagen Family. Cold Spring Harb. Perspect. Biol..

[5] Sadigursky D., Magnavita V.F.S., Sa C.K.C., Monteiro H.S., Braghiroli O.F.M., Matos M.A.A. (2022). Undenatured Collagen Type Ii for the Treatment of Osteoarthritis of the Knee. Acta Ortop. Bras..

[6] Zhou Y., Zhang Y., Dai H., Zhang Y., Fu Y. (2024). The potential of undenatured type II collagen against arthritis: A review. Collagen Leather.

[7] Dai M., Sui B., Xue Y., Liu X., Sun J. (2018). Cartilage repair in degenerative osteoarthritis mediated by squid type II collagen via immunomodulating activation of M2 macrophages, inhibiting apoptosis and hypertrophy of chondrocytes. Biomaterials.

[8] Choi B., Kim S., Lin B., Wu B.M., Lee M. (2014). Cartilaginous extracellular matrix-modified chitosan hydrogels for cartilage tissue engineering. ACS Appl. Mater. Interfaces.

[9] Rutgers M., Saris D.B., Vonk L.A., van Rijen M.H., Akrum V., Langeveld D., van Boxtel A., Dhert W.J., Creemers L.B. (2013). Effect of collagen type I or type II on chondrogenesis by cultured human articular chondrocytes. Tissue Eng. Part A.

[10] Jiang L.B., Su D.H., Liu P., Ma Y.Q., Shao Z.Z., Dong J. (2018). Shape-memory collagen scaffold for enhanced cartilage regeneration: Native collagen versus denatured collagen. Osteoarthr. Cartil..

[11] Pulkkinen H.J., Tiitu V., Valonen P., Jurvelin J.S., Lammi M.J., Kiviranta I. (2010). Engineering of cartilage in recombinant human type II collagen gel in nude mouse model in vivo. Osteoarthr. Cartil..

[12] Muhonen V., Narcisi R., Nystedt J., Korhonen M., van Osch G.J., Kiviranta I. (2017). Recombinant human type II collagen hydrogel provides a xeno-free 3D micro-environment for chondrogenesis of human bone marrow-derived mesenchymal stromal cells. J. Tissue Eng. Regen. Med..

[13] Wu Z., Korntner S.H., Mullen A.M., Skoufos I., Zeugolis D.I. (2021). In the quest of the optimal tissue source (porcine male and female articular, tracheal and auricular cartilage) for the development of collagen sponges for articular cartilage. Biomed. Eng. Adv..

[14] Xiao S., Huang X., He X., Chen Z., Li X., Wei X., Liu Q., Dong H., Zeng X., Bai W. (2025). Interactions between curcumin and fish/bovine-derived (type I and II) collagens: Preparation of nanoparticle and their application in Pickering emulsions. Food Chem..

[15] Akram A.N., Zhang C. (2020). Extraction of collagen-II with pepsin and ultrasound treatment from chicken sternal cartilage; physicochemical and functional properties. Ultrason. Sonochem.

[16] Cao H., Xu S.Y. (2008). Purification and characterization of type II collagen from chick sternal cartilage. Food Chem..

[17] Dai M., Liu X., Wang N., Sun J. (2018). Squid type II collagen as a novel biomaterial: Isolation, characterization, immunogenicity and relieving effect on degenerative osteoarthritis via inhibiting STAT1 signaling in pro-inflammatory macrophages. Mater. Sci. Eng. C Mater. Biol. Appl..

[18] Sewing J., Klinger M., Notbohm H. (2017). Jellyfish collagen matrices conserve the chondrogenic phenotype in two- and three-dimensional collagen matrices. J. Tissue Eng. Regen. Med..

[19] Zhang X., Adachi S., Ura K., Takagi Y. (2019). Properties of collagen extracted from Amur sturgeon Acipenser schrenckii and assessment of collagen fibrils in vitro. Int. J. Biol. Macromol..

[20] Avila Rodríguez M.I., Rodríguez Barroso L.G., Sánchez M.L. (2017). Collagen: A review on its sources and potential cosmetic applications. J. Cosmet. Dermatol..

[21] Chen C.-X., Zhang Y.-Y., Yang J., Yan M.-H., Jia Y., Jiang S. (2024). An overview of progress in the application of recombinant collagen in cosmetics. J. Dermatol. Sci. Cosmet. Technol..

[22] Xi C., Liu N., Liang F., Zhao X., Long J., Yuan F., Yun S., Sun Y., Xi Y. (2018). Molecular assembly of recombinant chicken type II collagen in the yeast Pichia pastoris. Sci. China Life Sci..

[23] Wang K., Yu S., Sun R., Xu K., Zhao X., Zhou J., Rao Y., Wang X. (2024). Biosynthesis of a Functional Fragment of Human Collagen II in Pichia pastoris. ACS Synth. Biol..

[24] Lioi M., Tengattini S., Bagatin F., Galliani S., Daly S., Massolini G., Temporini C. (2023). Development of a rapid, efficient, and reusable magnetic bead-based immunocapture system for recombinant human procollagen type II isolation from yeast fermentation broth. Anal. Bioanal. Chem..

[25] Qi Q., Yao L., Liang Z., Yan D., Li Z., Huang Y., Sun J. (2016). Production of human type II collagen using an efficient baculovirus-silkworm multigene expression system. Mol. Genet. Genom..

[26] Ala-Kokko L., Hyland J., Smith C., Kivirikko K., Jimenez S., Prockop D. (1991). Expression of a human cartilage procollagen gene (COL2A1) in mouse 3T3 cells. J. Biol. Chem..

[27] Zhou H., Mu Y., Ma C., Zhang Z., Tao C., Wang D.A. (2024). Rejuvenating Hyaline Cartilaginous Phenotype of Dedifferentiated Chondrocytes in Collagen II Scaffolds: A Mechanism Study Using Chondrocyte Membrane Nanoaggregates as Antagonists. ACS Nano.

[28] Rottmar M., Mhanna R., Guimond-Lischer S., Vogel V., Zenobi-Wong M., Maniura-Weber K. (2014). Interference with the contractile machinery of the fibroblastic chondrocyte cytoskeleton induces re-expression of the cartilage phenotype through involvement of PI3K, PKC and MAPKs. Exp. Cell Res..

[29] Xin W., Heilig J., Paulsson M., Zaucke F. (2015). Collagen II regulates chondroycte integrin expression profile and differentiation. Connect. Tissue Res..

[30] Fertala A. (2020). Three Decades of Research on Recombinant Collagens: Reinventing the Wheel or Developing New Biomedical Products?. Bioengineering.

[31] Revell C.K., Jensen O.E., Shearer T., Lu Y., Holmes D.F., Kadler K.E. (2021). Collagen fibril assembly: New approaches to unanswered questions. Matrix Biol. Plus.

[32] Aravamudhan A., Ramos D.M., Jenkins N.A., Dyment N.A., Sanders M.M., Rowe D.W., Kumbar S.G. (2016). Collagen nanofibril self-assembly on a natural polymeric material for the osteoinduction of stem cells in vitro and biocompatibility in vivo. RSC Adv..

[33] Huang J., Huang Z., Liang Y., Yuan W., Bian L., Duan L., Rong Z., Xiong J., Wang D., Xia J. (2021). 3D printed gelatin/hydroxyapatite scaffolds for stem cell chondrogenic differentiation and articular cartilage repair. Biomater. Sci..

[34] Hu X., Wu Z., Zhang Z., Yao H., Wang D.-A. (2024). Type II collagen scaffolds for tissue engineering. Commun. Mater..

[35] Chen R., Pye J.S., Li J., Little C.B., Li J.J. (2023). Multiphasic scaffolds for the repair of osteochondral defects: Outcomes of preclinical studies. Bioact. Mater..

[36] Zhou L., Gjvm V.O., Malda J., Stoddart M.J., Lai Y., Richards R.G., Ki-Wai Ho K., Qin L. (2020). Innovative Tissue-Engineered Strategies for Osteochondral Defect Repair and Regeneration: Current Progress and Challenges. Adv. Healthc. Mater..

[37] Kober L., Zehe C., Bode J. (2013). Optimized signal peptides for the development of high expressing CHO cell lines. Biotechnol. Bioeng..

[38] Attallah C., Etcheverrigaray M., Kratje R., Oggero M. (2017). A highly efficient modified human serum albumin signal peptide to secrete proteins in cells derived from different mammalian species. Protein Expr. Purif..

[39] Bender M.F., Li Y., Ivleva V.B., Gowetski D.B., Paula Lei Q. (2021). Protein and glycan molecular weight determination of highly glycosylated HIV-1 envelope trimers by HPSEC-MALS. Vaccine.

[40] Lebendiker M., Tsadok A., Amartely H., Some D. (2019). Characterization of Proteins by Size-Exclusion Chromatography Coupled to Multi-Angle Light Scattering (SEC-MALS). J. Vis. Exp..

[41] Anand S., Kamath S., Chuang L., Kasapis S., Lopata A.L. (2013). Biochemical and thermo-mechanical analysis of collagen from the skin of Asian Sea bass (*Lates calcarifer*) and Australasian Snapper (*Pagrus auratus*), an alternative for mammalian collagen. Eur. Food Res. Technol..

[42] Li C., Tian H., Duan L., Tian Z., Li G. (2013). Characterization of acylated pepsin-solubilized collagen with better surface activity. Int. J. Biol. Macromol..

[43] Zhu S., Yuan Q., Yin T., You J., Gu Z., Xiong S., Hu Y. (2018). Self-assembly of collagen-based biomaterials: Preparation, characterizations and biomedical applications. J. Mater. Chem. B.

[44] Sharma V., Sakhalkar U., Nadkarni P., Mishal R., Parandhaman D., Vichare K., Francis A., Khanna M., Kukreja M., Sharma A. (2024). Cytoprotective Effect of Growth Factors Derived from Platelets on Corticosteroid-Treated Primary Anterior Cruciate Ligament-Derived Stromal Cells and Chondrocytes. Cureus.

[45] Yu J., Liu Q., Zhang Y., Xu L., Chen X., He F., Zhang M., Yang H., Yu S., Liu X. (2025). Stress causes lipid droplet accumulation in chondrocytes by impairing microtubules. Osteoarthr. Cartil..

[46] Xuan H., Hu H., Geng C., Song J., Shen Y., Lei D., Guan Q., Zhao S., You Z. (2020). Biofunctionalized chondrogenic shape-memory ternary scaffolds for efficient cell-free cartilage regeneration. Acta Biomater..

[47] Wang C., Guo X., Fan M., Yue L., Wang H., Wang J., Zha Z., Yin H. (2024). Production of recombinant human type I collagen homotrimers in CHO cells and their physicochemical and functional properties. J. Biotechnol..

[48] Li J., Ke H., Lei X., Zhang J., Wen Z., Xiao Z., Chen H., Yao J., Wang X., Wei Z. (2024). Controlled-release hydrogel loaded with magnesium-based nanoflowers synergize immunomodulation and cartilage regeneration in tendon-bone healing. Bioact. Mater..

[49] Li Q., Yu H., Sun M., Yang P., Hu X., Ao Y., Cheng J. (2021). The tissue origin effect of extracellular vesicles on cartilage and bone regeneration. Acta Biomater..

[50] Klatt A.R., Paul-Klausch B., Klinger G., Kuhn G., Renno J.H., Banerjee M., Malchau G., Wielckens K. (2009). A critical role for collagen II in cartilage matrix degradation: Collagen II induces pro-inflammatory cytokines and MMPs in primary human chondrocytes. J. Orthop. Res..

[51] Lu Z.F., Doulabi B.Z., Huang C.L., Bank R.A., Helder M.N. (2010). Collagen type II enhances chondrogenesis in adipose tissue-derived stem cells by affecting cell shape. Tissue Eng. Part A.

[52] Wang K.Y., Jin X.Y., Ma Y.H., Cai W.J., Xiao W.Y., Li Z.W., Qi X., Ding J. (2021). Injectable stress relaxation gelatin-based hydrogels with positive surface charge for adsorption of aggrecan and facile cartilage tissue regeneration. J. Nanobiotechnol..

[53] Zhou Z., Lv C., Wang Y., Zhang B., Liu L., Yang J., Leng X., Zhao D., Yao B., Wang J. (2023). BuShen JianGu Fang alleviates cartilage degeneration via regulating multiple genes and signaling pathways to activate NF-kappaB/Sox9 axis. Phytomedicine.

[54] Lian C., Wang X., Qiu X., Wu Z., Gao B., Liu L., Liang G., Zhou H., Yang X., Peng Y. (2019). Collagen type II suppresses articular chondrocyte hypertrophy and osteoarthritis progression by promoting integrin β1−SMAD1 interaction. Bone Res..

[55] Li X., Yang S., Yuan G., Jing D., Qin L., Zhao H., Yang S. (2022). Type II collagen-positive progenitors are important stem cells in controlling skeletal development and vascular formation. Bone Res..

